# The Board of Health of the State of Georgia

**Published:** 1883-04

**Authors:** 


### THE BOARD OF HEALTH OF THE STATE OF GEORGIA.

Georgia is included in the list of States wise enough and prudent enough to provide a board of health for the protection of the health and lives of her citizens. At least the statute books indicate that she is possessed of this degree of wisdom and prudence; but, as we have on a previous occasion stated, it is merely a nominal advantage, for after two years of a precarious existence on scanty fare the Board, established in 1875, was starved out of being by the refusal of the legislature to appropriate the pittance necessary for its support, and the legislative act providing for its establishment and prescribing its duties has been a dead letter since that time.

In a report by ex-Justice Logan E. Bleckley, made in pursuance of appointment by resolution of the General Assembly, to examine the Code of Georgia as to omitted legislation enacted between 1873 and 1882, we find the following reference to the board, from which we infer that it is dead—as surely and as permanently dead as if its taking off had been devoid of the peculiar and irregular circumstances which attended its demise. But the remarks of this eminent jurist speak for themselves; they clearly set forth the present status of the board and, direct and consequential, are as follows:

"At present both these institutions, [the Geological Department and the Board of Health,] are (to say the least) in a state of legal torpor or suspended animation. The charters of their nativity—the statutes under which they were born, being unrepealed, they are still under the "old flag," but they lie in a protracted swoon for lack of an "appropriation." Moreover, as to the former, there has been a grant of administration upon its assets, which grant could only have been made upon the assumption that it was defunct. And this assumption has been followed by a peculiar *post mortem* resolution, a sort of capital sentence, *nunc pro tunc*—a judgment of death pronounced after execution. In the presence of documents apparently so ultimate, so final, it is not easy to accept for good law the teachings of the Code, that the Commissioner of Agriculture is entitled to employ a geologist to assist him in preparing a geological survey, or the still more apocryphal statement, a little further on, to the effect that there is a standing appropriation of ten thousand dollars a year to pay the expenses of such survey, together with the other enumerated objects of annual expenditure. If these provisions of the Code touching geological possibilities are sound, considerably more on the same subject might have been, perhaps ought to have been, introduced. On practical grounds, however, I am persuaded that it would have been safer to have left out what was inserted. The general spirit of the practical offers but spare encouragement to scientific zeal and devotion. Those capital prizes of science termed fossils are especially odious. They are stigmatized by the practical with the very superlatives of aversion and contempt. A fossil is lost matter, corresponding in physical reprobation to a lost soul in the spiritual. Stripped to its essence, it is time petrified by malediction; the past preserved by a curse. The law, intensely modern, always in league and sympathy with the present, always devoted to the practical, favors no fossils but its own. These it cherishes, sometimes too fondly, as priceless gems, inherited from ancestral opulence—the hoards of those grand old legal millionaires, the early sages.

"The vital relations of the Board of Health are more obscure than those of its scientific confrere. Whether the swoon in which it lies should be deemed equivalent to a formal demise, depends upon this novel question in legal pathology: Can a law—a law of health—die by parliamentary starvation, the same as by legislative bloodshed?

"Supposing the main question answered in the affirmative, and the Board itself remitted to its everlasting rest, must all its statutory adjuncts be treated as then extinct? For instance, are the county boards, which never had or expected any fiscal nourishment from the bosom of the State, a part of the general cadaver? And granting that the county boards also are no more, the ordinary and the coroner, as mere county officers, undoubtedly survive; and how stand their duties respecting vital statistics? Must the coroner still report every inquest to the ordinary? Must the ordinary still register all births, marriages and deaths that he hears of from authentic sources, upon payment or tender of his appointed stipend of five cents? The right answer as to services would seem to be this: If, according to the legislative scheme, properly construed, such services were intended to be partly dependent upon, and partly independent of, the rest of the system, the disruption of the system would be no dispensation from the services, in so far that their performance might still be practicable with the means left at command. But if the system was an entirety, its workings all inter-dependent as those of one organism, and their results not apportionable, then there could be no severance, and with the demise of the head perished all the members, and their every function, even to the remotest extremity. The range of decision is confined to these alternatives.

The pan-mortuary hypothesis is the one which has the sanction of official practice. I see not how it could be contested with any advantageous results. What can justly and forcibly be said is, that the dying at Board headquarters has been informal and irregular; but it is none the less real; and I am not aware that any vital interest of the departed, whether legal or sentimental, can be served by mere protest against the manner of the "taking off." There is not in law, any more than nature, a doctrine that death is void for irregularity."

